# A Novel DNA Methylation Signature as an Independent Prognostic Factor in Muscle-Invasive Bladder Cancer

**DOI:** 10.3389/fonc.2021.614927

**Published:** 2021-02-15

**Authors:** Zhijie Xu, Hemant Gujar, Guanghou Fu, Hamed Ahmadi, Sumeet Bhanvadia, Daniel J. Weisenberger, Baiye Jin, Parkash S. Gill, Inderbir Gill, Siamak Daneshmand, Kimberly D. Siegmund, Gangning Liang

**Affiliations:** ^1^Department of Urology, The First Affiliated Hospital, School of Medicine, Zhejiang University, Hangzhou, China; ^2^USC Institute of Urology and Norris Comprehensive Cancer Center, Keck School of Medicine, University of Southern California, Los Angeles, CA, United States; ^3^Department of Biochemistry and Molecular Medicine, Keck School of Medicine, University of Southern California, Los Angeles, CA, United States; ^4^Division of Hematology in Department of Medicine, Keck School of Medicine, University of Southern California, Los Angeles, CA, United States; ^5^Department of Preventive Medicine, Keck School of Medicine, University of Southern California, Los Angeles, CA, United States

**Keywords:** methylation and prognosis, bladder cancer (BC), survival analysis, MIBC, ROC, DNA methylation marker

## Abstract

**Background:**

Muscle-invasive bladder cancer (MIBC) accounts for approximately 20% of all urothelial bladder carcinomas (UBC) at time of diagnosis, and up to 30% of patients with non-muscle invasive UBC will progress to MIBC over time. An increasing body of evidence has revealed a strong correlation between aberrant DNA methylation and tumorigenesis in MIBC.

**Results:**

Using The Cancer Genome Atlas (TCGA) molecular data for 413 patients, we described a DNA methylation-based signature as a prognostic factor for overall survival (OS) in MIBC patients. By using a least absolute shrinkage and selection operator (LASSO) model, differentially methylated regions were first identified using multiple criteria followed by survival and LASSO analyses to identify DNA methylation probes related to OS and build a classifier to stratify patients with MIBC. The prognostic value of the classifier, referred to as risk score (RS), was validated in a held-out testing set from the TCGA MIBC cohort. Finally, receiver operating characteristic (ROC) analysis was used to compare the prognostic accuracy of the models built with RS alone, RS plus clinicopathologic features, and clinicopathologic features alone. We found that our seven-probe classifier-based RS stratifies patients into high- and low-risk groups for overall survival (OS) in the testing set (n = 137) (AUC at 3 years, 0.65; AUC at 5 years, 0.65). In addition, RS significantly improved the prognostic model when it was combined with clinical information including age, smoking status, Tumor (T) stage, and Lymph node metastasis (N) stage.

**Conclusions:**

The DNA methylation-based RS can be a useful tool to predict the accuracy of preoperative and/or post-cystectomy models of OS in MIBC patients.

## Introduction

### Background

Bladder cancer is the 5^th^ most common malignancy in the United States (1). Non-muscle invasive tumors (NMIBC) account for 80% of all bladder cancers, while muscle invasive tumors (MIBC) comprise the remaining 20% of bladder tumors. Interestingly, up to 30% of NMIBCs eventually progress to MIBCs (2). The current gold standard treatment for MIBC patients is neoadjuvant cisplatin-based chemotherapy (NAC) followed by radical cystectomy (3, 4), however, approximately 50% of these patients develop metastases within 2 years after diagnosis (5, 6). MIBC patients with localized disease show a 5-year survival rate of 60%, however, only 10% of MIBC patients with distant metastasis survive past 5 years. At present, pathologic staging, reported according to the TNM staging system, is most widely used to determine patient prognosis and to guide the choice of treatment following cystectomy (7, 8). However, even in patients with the same stage of MIBC there might be significant differences in prognosis and survival. In addition, prognostic tools to stratify risk and predictive biomarkers that facilitate selection of patients likely to respond to treatments such as bladder preservation, NAC, radical cystectomy, and adjuvant systemic therapies are essential to advance the field and personalize treatment. A variety of prognostic tools have been described for MIBC patients, including clinical features and tissue-based biomarkers (9–12), however, more specific markers and subtype classification are needed to aid in patient selection for treatment.

Aberrant DNA methylation is one of the most common epigenetic changes in all cancer types during tumorigenesis (including bladder cancer) and mediates tumor initiation, progression, invasion, metastasis, and drug resistance (13–19). DNA methylation is chemically stable and can be experimentally quantifiable, making it a promising tumor marker for bladder cancer detection, diagnosis, prognosis, and tumor recurrence (12, 20–24). Prior studies, including those from our team, have shown that bladder cancer-specific DNA methylation changes can be detected not only in tumor specimens, but also in urine sediments, and can be used as markers for diagnosis, prognosis and recurrence (13, 14, 18, 19, 25–28).

With the accelerated development of genome-wide technologies, new statistical algorithms and easily accessible public databases such as The Cancer Genome Atlas (TCGA) and the Gene Expression Omnibus (GEO) and the systematic collection of clinical, pathological, and biological data from various types of cancer (11, 29, 30) have offered a powerful validation pool for the identification of tumor markers.

In order to address the clinical need for accurate and reproducible measurements to identify patients with high mortality risk, we outline the development and validation of a practical and reliable DNA methylation classifier based on TCGA data that improves not only upon existing preoperative or pretreatment risk stratification, but also post cystectomy for MIBC patients. We demonstrate that this classifier predicts MIBC patients with high risk of mortality and increases precision in clinical decision making in current clinical practice.

## Methods

### Data Processing

DNA methylation (413 tumor samples and 21 paired normal-adjacent samples) and corresponding clinical information were retrieved from 413 bladder cancer patients in The Cancer Genome Atlas (TCGA) data portal. TCGA DNA methylation data and clinical data are publicly available and open access, therefore, no ethical issues were involved. Clinicopathologic features include sex, age, smoking history, T (tumor) stage, N (lymph node metastasis) status, M (distant metastasis) status, tumor grade, survival status, and survival time. We randomly divided the data into training and testing sets in a 2:1 training/testing format, in which the training group contains 276 tumors and 14 normal tissues, while the testing group contains 137 tumors and 7 normal tissues. The differences in clinicopathological characteristics including age, sex, smoking status, adjuvant treatment status, T stage, N stage, M stage, and tumor grade between training set and testing set were analyzed using chi-square tests. We repeated the random division 10 additional times to assess the stability of our model building process on the model prediction.

### Construction of the Risk Assessment Model

We filtered the features prior to building a risk prediction model. We first analyzed the training set DNA methylation data to identify differentially methylated probes between cancer and adjacent-normal tissues. Moderated t-tests were computed using the *limma* package in R software 3.6.1. Multiple testing p-value adjustment was performed using Benjamini and Hochberg’s method (FDR, false discovery rate), with a 0.05 threshold to identify differential DNA methylation. DNA methylation differences were characterized by logFC (|log_2_ fold change| > 1.5), the difference in log_2_ average β-value for each probe between bladder cancer and normal tissues. We further filtered the probes using the difference in average β-values between tumor and normal tissues, Tumor_β-value_-Normal_β-value_>0.4 and < −0.4, to select the hypermethylated and hypomethylated probes having the most clinical relevance. A heatmap (**Figure 2A**) displaying the clustering of samples and filtered probe sets with columns ordered by tissue type and rows by fold-change. The probes identified to be differentially methylated by the above algorithms were selected to the next stage of study.

Second, we performed survival analysis using a univariate Cox model to investigate the relationship between the DNA methylation level of each differentially methylated probe (DMP) and patient overall survival (OS). Hazard ratios (HRs) and p-values of each hypermethylated (n = 341) and hypomethylated (n = 26) probe were calculated to identify potential survival-related DMPs. “Protective” probes were defined as DMPs with HR for death <1, while “risky” probes were defined as DMPs with HR for death >1. We selected survival-related DMPs with Cox P < 0.05 and performed LASSO Cox regression to build a model to predict OS. We ultimately constructed a seven-probe classifier to predict OS of MIBC patients. In order to quantify the risk of each patient, a standard form of risk score (RS) for each patient was calculated by combining the DNA methylation β-value of each probe (β_i_) and LASSO coefficients (L_i_), $\text{Risk Score}={\sum_{i=1}^{7}\beta }_{i}\times \;L_{i}$. The sensitivity and specificity of the classifier in predicting patient survival was analyzed using a time-dependent ROC curve. The best cutoff for dividing the patients into high- or low-risk groups was set at the value in which the ROC curve achieved optimum for predicting 5-year OS of the training set. We validated this RS model in the testing set. The *glmnet* package in R computing language was used for the LASSO Cox regression analysis and the *TimeROC* package was used for survival analyses.

### Survival Analysis

Kaplan-Meier curves and the log-rank test were used to distinguish clinical prognostic features using the TCGA bladder cancer cohort. Kaplan-Meier curves for the clinicopathologic features (T stage and N stage) were further evaluated after stratifying subjects into high- and low-risk groups, as determined by the DNA methylation RS. Univariate Cox regression was performed to investigate the relationship of clinicopathological features (age, sex, smoking status, adjuvant treatment [pharmaceutical], T stage, and N stage), and risk score with bladder cancer patient OS. Due to the large number of patients missing information on adjuvant treatment, we used a missing data indicator variable approach to model all patients. We used two indicator variables; the first indicator variable was 1 if treated and 0 otherwise (not-treated or missing treatment information) and the second indicator was 1 if treated or not-treated and 0 if missing treatment information. For this scheme, the hazard ratio estimate for the first variable measures the risk in patients treated with adjuvant therapy compared to not-treated patients and the estimate for variable two measures the risk in patients not treated relative to patients with no information recorded.

A multivariate Cox regression model was next performed to test whether the prognostic value of the RS classifier is independent of clinical features that are significant in univariate analysis. To compare the accuracy of the prognostic classifier with clinicopathologic features in predicting bladder cancer patient outcome, receiver-operator characteristic (ROC) curves were generated for the RS alone, all clinical information (age + smoking history + T stage + N stage), and RS combined with clinical information. The ROC area under the curve (AUC) values were calculated and compared. In addition, in order to evaluate the joint prognostic value of risk score and clinical feature, the RS was integrated with T stage or N stage respectively, using logistic regression.

More importantly, as to better predict survival probability of bladder cancer patients, a nomogram that integrated both the RS model and clinical features was designed using the R-based *rms* package. First, we applied the cph function to fit the risk ratio model. Second, we used the function nomogram to draw the nomogram. Nomogram efficiency was validated by drawing calibration curves and calculating the concordance index (C-index).

## Results

### Data Acquisition and Patient Clinicopathological Features of the TCGA Bladder Cancer Dataset

**Figure 1:**
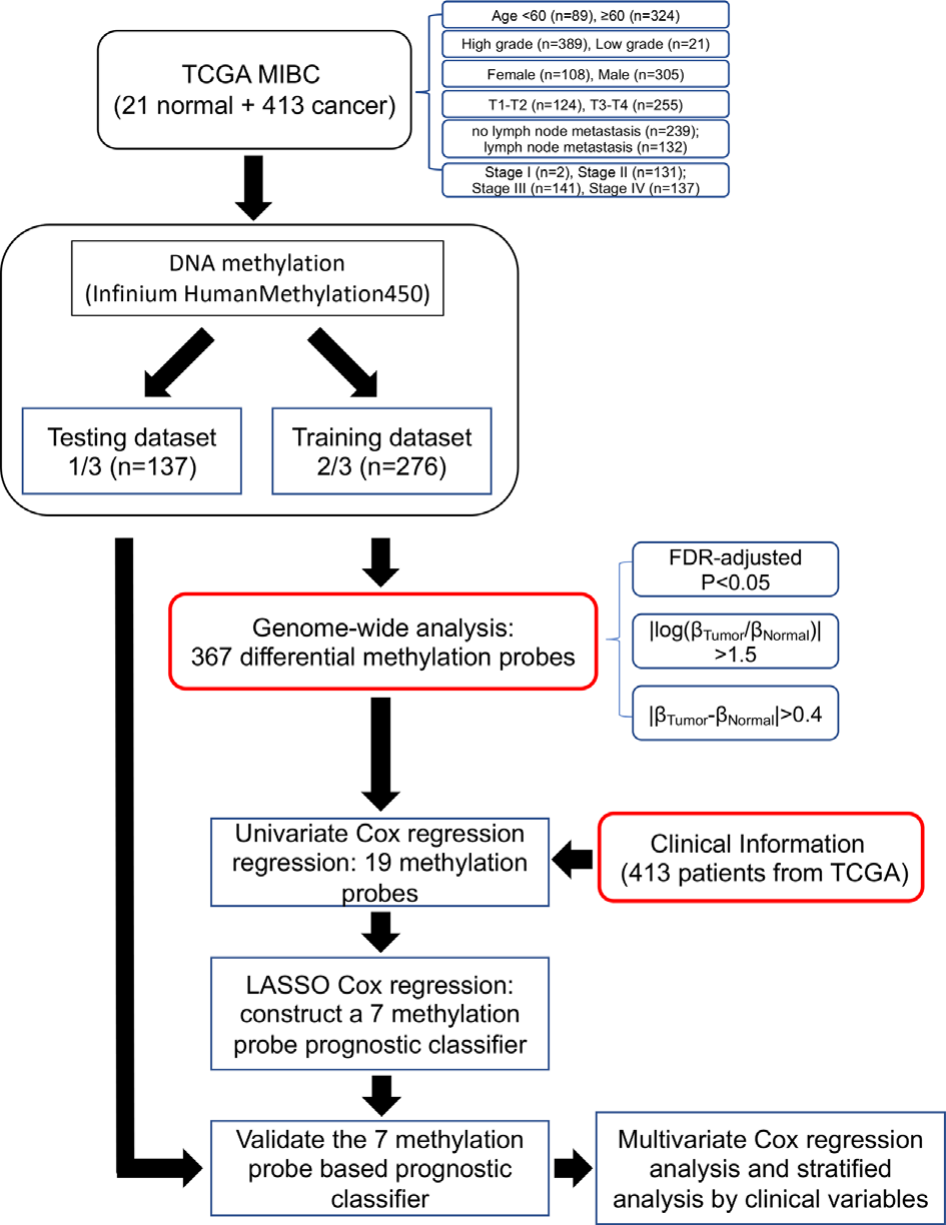
Flow chart indicating study design. TCGA BLCA data were used in the study and were divided in training and testing groups. Out of 367 differentially methylated probes, we obtained 19 probes from univariate Cox regression analysis. Of these, we constructed a seven-probe prognostic classifier based on a LASSO-regularized Cox model.

Bladder cancer molecular and corresponding clinical data were downloaded from the TCGA Data Portal (portal.gdc.cancer.gov/). DNA methylation data from the Illumina Infinium Human Methylation450 platform, (available on 413 tumor samples and 21 normal-adjacent samples) was downloaded with UCSC Xena (https://xena.ucsc.edu/) (31, 32). Patient demographic and clinical data, including age, gender, smoking status, adjuvant treatment, pathological stage, tumor grade, and survival data, were extracted. Data from 413 TCGA bladder patients were randomly divided into training (14 normals and 276 tumors) and testing groups (7 normals and 137 tumors) and patient clinicopathological features compared (**Table 1**). Please note, because of limited cases or information, TURBT (a transurethral resection of bladder tumor) and radiotherapy information in these patients were excluded for further analysis (**Table 1**). As shown in **Table 1**, there was no significant distribution bias between the training and testing groups with respect to age, sex, smoking status, pathological stage, TMN stage, and tumor grade and survival (p > 0.05). The selection procedure for identification of the prognostic DNA methylation signature is presented in **Figure 1**.

**Table 1:** Clinicopathological features for the 413 TCGA bladder cancer patients in the training set and testing set.

Characteristics	TCGA cohort		P-value^a^
	Training set n = 276	Test set n = 137	
**Age (years)**			
<60	63 (22.8%)	26 (19.0%)	0.3705
≥60	213 (77.2%)	111 (81.0%)	
**Sex**			
Female	74 (26.8%)	34 (24.8%)	0.6642
Male	202 (73.2%)	103 (75.2%)	
**Smoke**			
**** Yes	188 (68.1%)	93 (67.9%)	0.4293
**** No	70 (25.4%)	39 (28.5%)	
**** NA	18 (6.5%)	5 (3.6%)	
**T (Tumor) stage**			
Organ confined (T1,T2)	79 (28.6%)	45 (32.8%)	0.4748
Extravesical (T3, T4)	176 (63.8%)	79 (57.7%)	
NA	21 (7.6%)	13 (9.5%)	
**N (Lymph node metastasis) stage**			
N0	159 (57.6%)	80 (58.4%)	0.3678
N1	29 (10.5%)	19 (13.9%)	
N2	49 (17.8%)	27 (19.7%)	
N3	7 (2.5%)	1 (0.7%)	
NA	32 (11.6%)	10 (7.3%)	
**M (distant metastasis) stage**			
M0	130 (47.1%)	67 (48.9%)	0.9043
M1	7 (2.5%)	4 (2.9%)	
NA	139 (50.4%)	66 (48.2%)	
**TMN stage**			
I	1 (0.4%)	1 (0.7%)	0.1812
II	86 (31.2%)	45 (32.8%)	
III	101 (36.6%)	40 (29.2%)	
IV	88 (31.9%)	49 (35.8%)	
NA	0 (0%)	2 (1.5%)	
**Tumor grade**			
Low	16 (5.8%)	5 (3.7%)	0.6457
High	258 (93.5%)	131 (95.6%)	
NA	2 (0.7%)	1 (0.7%)	
**Median survival (days)**	1005	1036	0.914

a Pearson chi-square test or Fisher exact test was used for comparison between subgroups. NA, not available.

### Construction of a Prognostic DNA Methylation Classifier

**Figure 2:**
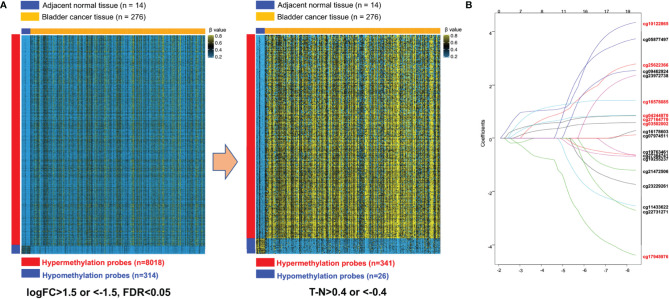
Construction of the seven-probe classifier. **(A)** Two heatmaps showing DNA methylation of the 8,332 and 367 differential methylation probes between adjacent-normal and tumor tissues in training set. **(B)** LASSO Cox regression coefficient profiles of the 19 survival-related probes were narrowed down into the final set of seven survival-related probes (marked as red).

**Figure 3:**
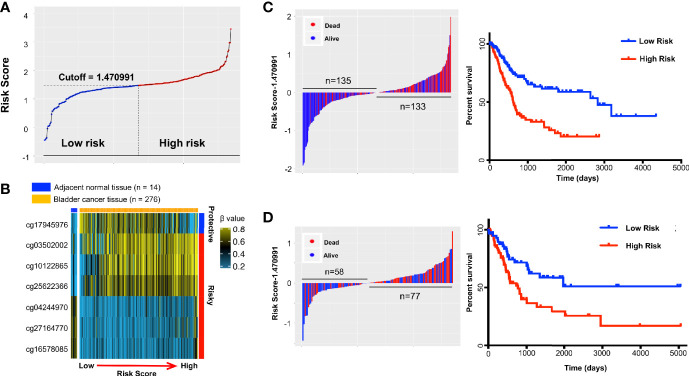
The distribution of risk score (RS) and Kaplan-Meier survival based on the classifier in the training and testing sets. A seven-probe classifier was used to calculate the RS. **(A)** The distribution of risk scores in training set. **(B)** Heatmap showing DNA methylation profiles of the seven probes in the training set sorted by adjacent-normal and tumors and by increasing RS. **(C)** Training set and **(D)** Testing set. Left panel: risk score distribution of the seven-probe classifier and patient survival status. Right panel: Kaplan-Meier patient survival analysis for the patients.

We developed and validated a prognostic DNA methylation-based classifier by dividing the TCGA samples into training (Normal = 14, Tumor = 276) and testing groups (Normal = 7, tumor = 137). Using thresholds of |log_2_FC|>1.5, and FDR-adjusted p < 0.05, we identified a total of 8,332 Differentially Methylated Probes (DMPs) in supervised analysis (8,018 hypermethylated and 314 hypomethylated probes) between normal and tumor samples in the training group. Using a more stringent filtering scheme of setting the DMP threshold to |βtumor-βnormal|>0.4, we identified 367 DMPs (341 hypermethylated and 26 hypomethylated probes) (**Figure 2A** and **Supplemental Figure S1** for unsupervised analysis). Next, we performed univariate Cox regression analysis to enrich for DMPs related to clinical outcome, and we then calculated hazard ratios (HR) for each probe. Probes with HR <1 were defined as *protective*, while probes with HR >1 were defined as *risky*. By selecting the probes with p-value <0.05, we identified 19 probes that were significantly correlated with MIBC patient survival (**Table 2**). Contained in this list are 7 *protective* probes and 12 *risky* probes (**Table 2**). In order to further reduce the number of probes for potential clinical application as prognostic markers, we used a LASSO Cox regression model on the set of 19 probes and calculated regression coefficients for each probe (**Figure 2B**, **Table 2**). According to this model, we calculated a risk score (RS) for each patient based on individual DNA values of seven probes (marked as red in **Figure 2B** and **Table 2**). The RS cutoff point for dividing high-risk and low-risk patients was calculated as 1.47 and was generated according to the optimum sensitivity (66.3%) and specificity (74.2%) from the ROC curves for predicting 5-year patient survival (**Figure 3A**). Patients with a RS ≥ 1.47 were classified as *high-risk*, while the remaining patients were classified as *low-risk* (**Figure 3A**). In addition, the patients with high RS tended to display DNA hypermethylation in tumors at *risky* probes and DNA hypomethylation at *protective* probes (**Figure 3B**). In addition, we determined the DNA methylation and gene expression status for these probes using the TCGA BLCA dataset. Interestingly, the seven *MEIS1* all display DNA hypermethylation in bladder cancer and are negative correlated with *MEIS1* expression. Alternatively, all seven *OTX1* probes also display DNA hypermethylation but are positively correlated with *OTX1* expression. We also found that DNA methylation of probes for *CPC6*, *SLAMF7*, *INTU*, and *LOC338758* are also positively correlated with their expression status (See **Supplemental Figure S2**). This suggests that we not only identified DNA methylation markers but also potential indicators of gene expression status.

**Table 2:** The DNA methylation based 19-probe panel that is significantly associated with MIBC patient survival in training set and the coefficients based on LASSO Cox regression analysis.

Symbol	Chromosome location	Gene name	DNA location	Univariate Cox regression analysis			LASSO coefficient
				HR	95% CI	P value	
**Protective Probes**							
cg17945976	Chr2:66520674-66520966	MEIS1	Gene body	0.45	0.23–0.87	0.017	−0.750495
cg23972738	Chr2:66520543-66520578	MEIS1	Gene body	0.48	0.26–0.89	0.019	.
cg22731271	Chr2:66520543-66520578	MEIS1	Gene body	0.5	0.28–0.88	0.015	.
cg16178603	Chr2:66667100-66667102	MEIS1	Gene body	0.51	0.27–0.94	0.031	.
cg09462924	Chr2:66519796-66520280	MEIS1	Gene body	0.51	0.27–0.94	0.031	.
cg05877497	Chr2:66521256-66521519	MEIS1	Gene body	0.52	0.27–0.98	0.042	.
cg11433622	Chr2:66521040-66521095	MEIS1	Gene body	0.52	0.28–0.94	0.03	.
**Risky Probes**							
cg27364741	Chr2:63134302-63135304	OTX1	Gene body	2.7	1.1–7	0.036	.
cg23229261	Chr2:63137299-63137651	OTX1	3'UTR	2.7	1.2–5.9	0.012	.
cg21472506	Chr2:63283936-63284147	OTX1	3'UTR	2.8	1.3–6	0.008	.
cg07974511	Chr2:63282514-63283122	OTX1	Gene body	2.9	1.1–7.6	0.03	.
cg03502002	Chr18:74961556-74963822	GALR1	1stExon;5'UTR	3	1.2–7.3	0.017	0.3186102
cg19763461	Chr2:63285949-63287097	OTX1	3'UTR	3.1	1.3–7.8	0.014	.
cg10122865	Chr2:63283936-63284147	OTX1	3'UTR	3.4	1.5–7.4	0.0028	1.0489578
cg25622366	Chr2:63281034-63281347	OTX1	Gene body	3.7	1.3–10	0.013	0.7313726
cg04244970	Chr1:160708825-160709105	SLAMF7	TSS200	3.9	1.3–12	0.017	0.5908991
cg27164770	Chr13:94891444-94891446	GPC6	Gene body	4.3	1.5–13	0.0083	0.5485644
cg16578085	Chr4:127771713-127771715	INTU	Gene body	4.5	1.2–17	0.026	1.1291202
cg10255237	Chr12:90150416-90150873	LOC338758	Gene body	4.8	1.1–22	0.04	.

HR, harzard ratio; CI, confidence interval; NA, no information.

### Performing and Validating the DNA Methylation Classifier-Based Risk Score (RS) for Prognostic Prediction in Bladder Cancer Patients

The relationship between RS and survival of bladder cancer patients in the training group is illustrated using Kaplan-Meier analysis. By design, patients with higher RS had a significantly worse overall survival (OS) than those with lower RS (**Figure 3C**). The median survival in high-risk group was 615 days *versus* 2,828 days in low-risk group (hazard ratio [HR] 2.502, 95% CI 1.731–3.617; **Figure 3C**). The prediction was validated in the testing group with a 2-fold increased risk of death for the high *vs* low RS (HR = 2.154, 95% CI 1.304–3.558; p = 4E-03; **Figure 3D**). In addition, high RS patients also tended to further gain DNA methylation in tumors at risky probes and lose DNA methylation at the protective probe in testing group (**Supplemental Figure S3A**) and are very similar to training group (**Figure 3B**). As the majority of follow-up data was within 5 years, ROC curves were used to assess prognostic power using OS data at 3 and 5 years after diagnosis. The ROC AUC values ranged in the training dataset from 0.70 to 0.72 and in the testing set from 0.68 to 0.65 (**Supplemental Figures S3B, C**).

### Repeated Sampling for Construction of Prognostic Classifier

To assess the robustness of our feature selection and prognostic classifier, we repeated 10 times the random sampling of data into training and testing groups (**Supplemental Table 1**). In the 10 training sets, an average of 20 features (range: 16–27) passed the differential DNA methylation filters and predicted patient survival. Of these, 62 features were unique. All 19 of our survival-predicting features in **Table 2** appeared in this superset of 62. Applying the LASSO model for each of the 10 feature-filtered training groups resulted in models that ranged in size from five to 12 features. Features mapping to the gene *MEIS1* appeared in all 10 models, features from *OTX1* appeared in nine models, and features from *INTU* and *SLAMF7* appearing in eight. Features from genes *GPC6* and *GALR1* appeared in six of the 10 models. Our LASSO model reported in **Table 2** selected seven features that mapped to five of these six genes (*MEIS1*, *OTX1*, *GALR1*, *SLAMF7*, *GPC6*), supporting the robustness of the features and genes reported. We computed 3-year and 5-year RS-AUC for each of the 10 testing group ROC curves. The average 3-year AUC of 0.61 (95% CI 0.50–0.76) and 5-year AUC of 0.63 (95% CI 0.49, 0.77) suggested the RS model can predict OS in independent datasets (**Supplemental Figure S4**).

### DNA Methylation-Based Risk Score (RS) Is a Prognostic Indicator for Patient Survival

**Figure 4:**
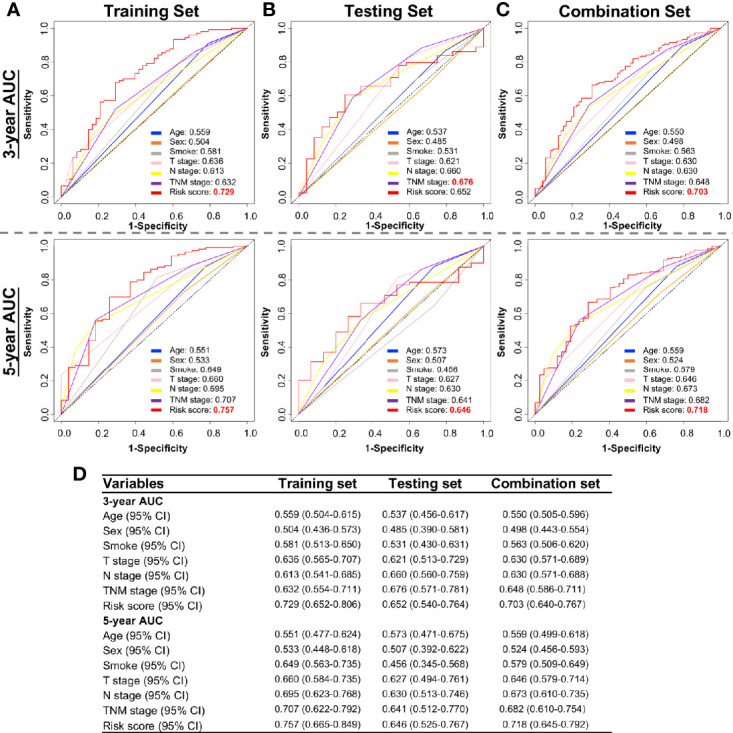
Time-dependent ROC curves showing the prognostic accuracy for the prognostic classifier (seven-probe-based classifier) and clinicopathological features in the TCGA BLCA cohort. Sex, Smoking status, T stage (T1–T4), N stage (N0–N3), and risk score. ROC curves at 3 and 5 years after diagnosis in testing set. ROC, receiver operator characteristic; AUC, area under the curve. **(A)** Training set; **(B)** Testing set; **(C)** Combination set. **(D)** The 95% confidence interval (CI) of RS and clinicopathological features in training set, testing set, and combination sets.

**Figure 5:**
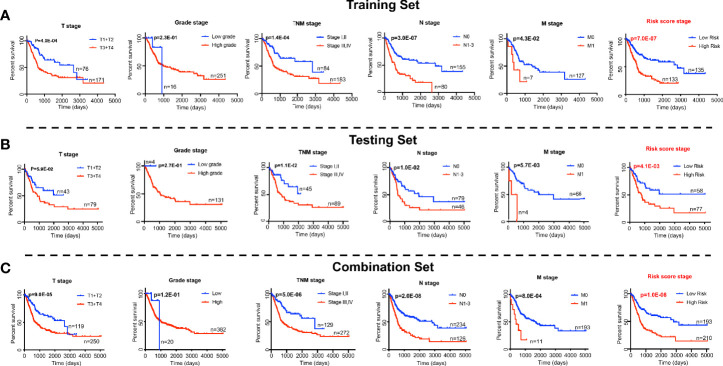
Log-rank p-values testing significance in overall survival for clinical features (the T stage, grade stage, N stage, and M stage) and RS based the prognostic classifier (seven-probe-based classifier) in the TCGA Bladder Cancer cohort. **(A)** Training set; **(B)** Testing set; **(C)** Combination set. The difference between the two curves were determined by the two-sided log-rank test.

We next tested whether the DNA methylation-based RS is a superior and/or independent indicator of mortality amongst MIBC patients by comparing individual clinicopathologic features including age, sex, smoking status, T stage, and N stage (**Table 1**). Time-dependent ROC curves were applied to compare the predictive accuracy between the RS and the other independent clinical factors for 3- and 5-years after diagnosis (**Figure 4**). As validated in the testing set, we found our DNA methylation-based RS had the highest AUC values at both time points, not only in the training set, but also in the testing and combination sets (**Figures 4A–D**). We also compared our DNA methylation-based RS to clinical features (Tumor stage: organ confined [T1+T2] with extravesical [T3+T4]; grade stage: low and high; N stage: N0 with N1-3; M stage: M0 with M1) using Kaplan Meier analysis. The RS is a prognostic indicator not only in training but also in testing and combination groups (**Figures 5A–C**).

We next tested whether a multivariable model for prognosis improves upon single-variable models by evaluating the independent contribution of the DNA methylation-based RS in a model that contained important clinicopathologic features (Sex, Age, Smoking history, T stage, and N stage) and adjuvant therapy for bladder cancer patients. To accomplish this, we performed Cox regression to identify correlations between each clinical feature, adjuvant treatment, the RS, and patient survival in our test dataset. After univariate and multivariate analysis, the DNA methylation-based classifier remained a powerful and independent prognostic indicator in the training and testing groups, not only in univariate analysis, but also in multivariate analysis, which is dependent on multiple clinical features including patient age and N stage, and is therefore more meaningful for reliable clinical prediction (**Table 3**). Although adjuvant treatment showed a protective effect on survival (HR = 0.68 in the combination set, 95% CI 0.45–1.03), it did not achieve statistical significance at the p < 0.05 level and was not included in the prediction model.

**Table 3:** Cox regression analyses of prognostic factors and overall survival of patients in the training, testing, and combination sets.

Variables	Categories	Univariate analysis		Multivariate analysis	
		HR (95% CI)	P value	HR (95% CI)	P value
**Training set, n = 276**					
Age	≥60/<60years	2.491 (1.328–4.673)	0.004474	2.067 (1.096–3.900)	0.024990
Sex	Male/female	0.995 (0.634–1.562)	0.984080		
Smoke	Yes/No	1.768 (1.079–2.900)	0.023846	1.694 (1.032–2.781)	0.037043
T stage	T3-T4/T1-T2	2.182 (1.291–3.688)	0.003565	1.718 (0.990–2.982)	0.054499
N stage	N1-N3/N0	2.487 (1.656–3.736)	0.000011	1.846 (1.198–2.846)	0.005458
Risk score (RS)	High/low	2.684 (1.744–4.131)	0.000007	2.200 (1.412–3.428)	0.000494
Adjuvant treatment (pharmaceutical)	Yes/(No or missing)	0.710 (0.414–1.218)	0.213501		
	recorded/missing	0.725 (0.481–1.093)	0.124510		
**Testing set, n = 137**					
Age	≥60/<60years	1.963 (0.838–4.598)	0.120381		
Sex	Male/female	0.632 (0.358–1.118)	0.114841		
Smoke	Yes/No	0.940 (0.527–1.675)	0.833041		
T stage	T3-T4/T1-T2	1.837 (0.943–3.58)	0.073894		
N stage	N1-N3/N0	2.030 (1.179–3.496)	0.010702	1.916 (1.110–3.310)	0.030489
Risk score (RS)	High/low	2.024 (1.132–3.62)	0.017406	1.903 (1.062–3.408)	0.019641
Adjuvant treatment (pharmaceutical)	Yes/(No or missing)	0.640 (0.326–1.254)	0.193260		
	recorded/missing	0.769 (0.416–1.422)	0.403125		
**Combination set, n = 413**					
Age	≥60/<60years	2.264 (1.366–3.752)	0.001526	1.863 (1.118–3.103)	0.016846
Sex	Male/female	0.843 (0.593–1.199)	0.342767		
Smoke	Yes/No	1.376 (0.948–1.996)	0.093329		
T stage	T3-T4/T1-T2	2.040 (1.351–3.080)	0.000697	1.533 (1.000–2.350)	0.049950
N stage	N1-N3/N0	2.304 (1.666–3.186)	0.000000	1.849 (1.319–2.593)	0.000366
Risk score (RS)	High/low	2.412 (1.711–3.400)	0.000000	2.039 (1.438–2.891)	0.000064
Adjuvant treatment (pharmaceutical)	Yes/(No or missing)	0.678 (0.446–1.030)	0.068814		
	recorded/missing	0.747 (0.533–1.048)	0.090917		

HR, hazard ratio; CI, confidence interval.

### Combination of the DNA Methylation-Based Risk Score (RS) With Clinical Features Adds Value Over Clinicopathologic Features Along

**Figure 6:**
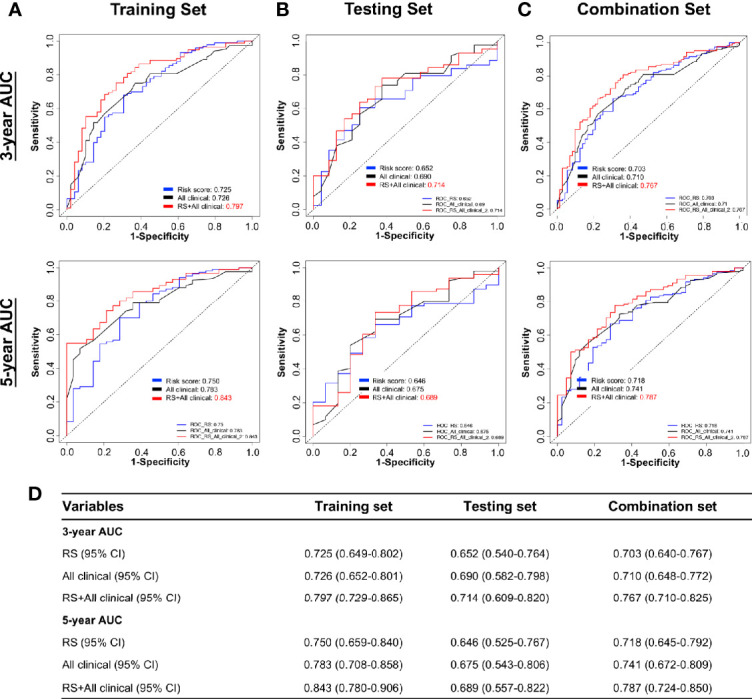
Performance of combining of multiple clinicopathological features with risk score in time-dependent ROC curves for the prognostic accuracy in the TCGA BLCA cohort. Risk Score (RS), all clinicopathological features: age, smoking status, T stage (T1–T4), N stage (N0–N3), and combination of all clinicopathological features and risk score. ROC curves at 3 and 5 years after diagnosis in training **(A)**, testing **(B)**, and combination set **(C)**. **(D)** The 95% CI of AUC in each data set. ROC, receiver operator characteristic; AUC, area under the curve.

The RS classifier is established based on tumor DNA methylation status, is independent of other MIBC clinicopathological features, and can predict patient survival at pre-operative status if biopsy- or TURBT-derived DNA samples can be obtained before cystectomy. We next evaluated whether the RS adds prognostic value to the current system that mostly depends on clinicopathological features. Indeed, by combining RS with the important clinicopathologic features available in TCGA dataset (age, smoking status, T stage, N stage), the calculated ROC AUC values showed a boost in performance for the combination model (**Figure 6**). The RS shows the highest specificity and sensitivity across the widest range of cutoffs (AUC) in training, testing, and combination sets over 3 and 5-year survival timelines (**Figures 6A–D**).

**Figure 7:**
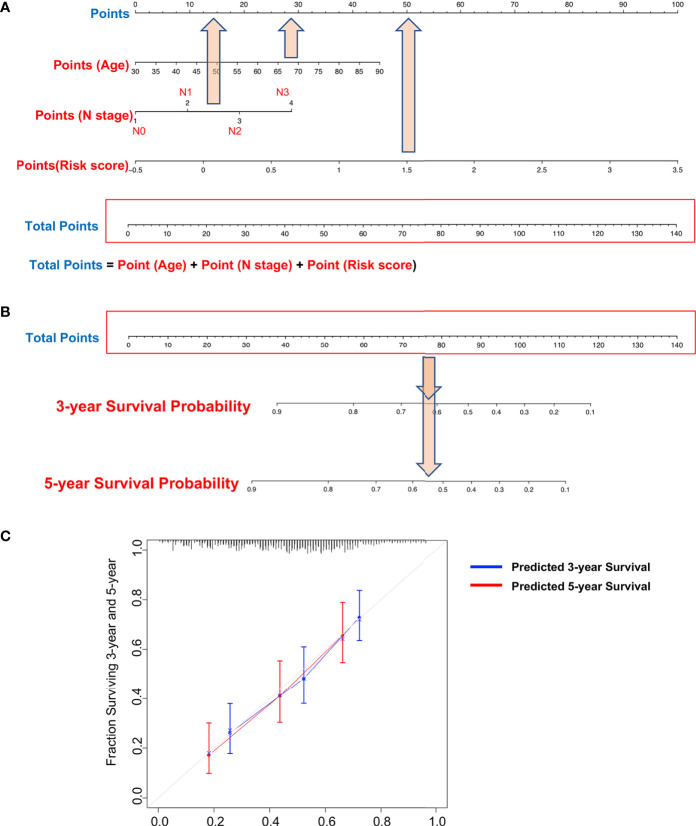
Nomograms to predict 3-year and 5-year survival probability in bladder cancer. **(A)** Total points were obtained by summing the corresponding points of each individual covariate (age, lymph node status, and risk score) on the points scale. **(B)** Total points were directly converted to particular 3-year and 5-year related survival probabilities. **(C)** Calibration plot for the nomogram. Dashed lines indicate the ideal reference line where predicted probabilities would match the observed proportions. Dashed lines represent nomogram-predicted probabilities grouped for each of the two groups, along with the respective confidence Intervals.

Our DNA methylation-based RS classifier also significantly subdivides short and long patient survival into high-risk and low-risk groups together with common clinicopathologic features (T stage and N stage) (**Supplemental Figures S5A, B**). Due to limited sample sizes within categories of the individual clinical variables, these figures show results for all patients combined (training and testing sets). These results suggest that combining RS and patient clinical features further improves prognostic accuracy. Therefore, we established a nomogram to integrate both the DNA methylation-based RS classifier and clinical features to predict survival probability in MIBC patients who had undergone surgical resection. However, besides RS, only age and lymph node metastasis categories have potential predictive value after multivariate analysis of the TCGA survival data (**Table 3**). Thus, we reduced our nomogram to include only age, lymph node metastasis, and RS (**Figures 7A, B**). For example, a 70-year-old bladder cancer patient with a single positive lymph node in the true pelvis (N1) and a RS of 1.5 has a total score of 90 points (= 30 + 10 + 50 points). The patient’s survival probability at 3 years would be 45% and 35% survival probability at 5 years. Calibration plots showed that prediction of 3-year and 5-year survival probabilities were highly similar to observed proportions (**Figure 7C**).

The RS is dependent on the DNA methylation status of seven probes. Interestingly, some of these probes are putative functional markers and were highly correlated to their gene expression in TCGA MIBC data (**Supplemental Figures S6A–F** and **Figure S6**). Probes cg25622366 and cg10122865 are examples of such functional markers, located in transcribed region (gene body) of *OTX1* and scientifically positively correlated with OTX1 gene expression (DNA hypermethylation of these probes along with increased of OTX1 expression) (**Supplemental Figures S6B, C**). This finding is also supported by our previous studies regarding the positive correlation between gene body DNA methylation and gene expression (33, 34).

## Discussion

In this study, the RS based on a DNA methylation signature of seven probes is independent of MIBC patient clinical features and shows a possible improved prognostic value for 3- and 5-year survival after diagnosis. As an independent predictor, the RS outperforms most clinicopathologic features, such as T stage, N stage, M stage. Most importantly, combining the RS algorithm with clinicopathologic features improves the predicted score for patient survival, suggesting that clinical data and RS provide independent and complementary prognostic information.

The DNA methylation-based RS is independent of the most important clinical and clinicopathologic features, such as age, sex, smoking status, T stage, and N stage, for predicting prognosis and can be used preoperatively by analyzing tumor biopsies or tumor-derived, cell-free DNA in patient urine prior to clinical intervention. We also took advantage of the nomogram we developed in this study that merges our DNA methylation-based RS with patient age and lymph node metastasis status to obtain improved prognostic value for MIBC patients. Please note that a bladder cancer nomogram (9) has been previously developed to predict recurrence risk after radical cystectomy, but is dependent on multiple clinical features such as patient age, gender, time from diagnosis to surgery, pathologic tumor stage and grade, tumor histologic subtype, and regional lymph node status (9), while our nomogram is only dependent on the DNA methylation-based RS, patient age, and lymph node status. Our approach improves upon not only preoperative but also post-cystectomy patient counseling along with clinicopathologic features and better identifies candidate patients who require more aggressive management.

Recently, a biomarker panel comprised of six genes and MIBC clinical features was identified for bladder cancer patient survival prediction based on TCGA data (24). The model was restricted to DNA methylation changes associated with gene expression alterations, eliminating many DNA methylation-based markers that show superior survival outcome prediction. Further, the model was estimated using the entire dataset, leading to overestimation of model accuracy. Still, our risk score HR of 2.0 [95% CI 1.1–3.6] estimated in our independent testing set is stronger than their risk score HR of 1.5 [95% CI 1.3–1.7] estimated using all patients. Using all patients combined, our risk score HR is 2.1 [95% CI 1.5–2.9].

There are two potential clinical implications to help patient decision-making using our RS: 1) Selection of patients for bladder preservation therapy and 2) Selection of radial cystectomy with or without neoadjuvant cisplatin-based chemotherapy (NAC). Bladder-preservation therapy for MIBC patients has shown benefit for MIBC patients in maintaining normal bladder function (35–37). Currently, many MIBC patients are candidates for bladder preservation with trimodal therapy (maximal TURBT, chemotherapy, and radiotherapy) (38, 39). Our RS may potentially help to select targeted patients for preservation therapy in clinical decision making, such as candidates with high RS should avoid bladder-preservation therapy but radical cystectomy.

The gold standard treatment for MIBC patients has classically been radical cystectomy (RC) (3), however, approximately 50% of these patients develop metastases within 2 years (5, 6). With evidence showing an overall survival benefit with cisplatin-based chemotherapy prior to surgery for MIBC patients, cisplatin-based NAC is now the standard of care (4, 40). This benefit is the greatest in patients with complete pathological response or down-staging, however, only approximately 40% of patients show this level of drug response to NAC. A substantial proportion of patients are subjected to the morbidity and side effects of chemotherapy without certain clinical benefit (4, 40). Additionally, chemotherapy treatment delays time to cystectomy in patients that are not responsive to NAC administration, a delay that is associated with poorer outcomes (4, 40). NCCN and EAU guidelines recommend (41, 42) adjuvant chemotherapy and/or radiotherapy after RC as the standard treatment for pT3-pT4 stage tumors, or tumors with positive nodes or positive margins not including pT2 patients. Based on our findings, if RS can be obtained from their biopsy samples, we suggest that pT2N0M0 patients with high RS (poor prognosis) should also be considered to receive adjuvant treatment as cisplatin-based chemotherapy or radiotherapy if no NAC is administered. Testing whether RS can predict NAC response in patients is very important, however, only 10 of 413 patients (2.4%) in the TCGA dataset have NAC treatment information. We recommend RS testing in TURBT samples prior to NAC treatment in future studies.

As we mentioned before, cancer detection and surveillance by identification of altered DNA methylation is quite robust for the advantage of DNA’s inherent stability compared with RNA or protein-based biomarkers. Nowadays, more and more studies revealed that the use of DNA methylation is an extremely sensitive strategy for detection, prediction of cancer risk, and prognosis. We have developed a DNA methylation signature based on seven probes for MIBC patient prognosis. These biomarkers can be detected and quantified not only in tumor specimens and biopsies, but also tumor-derived, cell-free DNA (cfDNA) present in urine and blood to predict patient outcome. Indeed, we have successfully developed non-invasive, urine-based DNA methylation assays for bladder cancer diagnosis and tumor recurrence (13, 25, 28). Although there is concern that DNA methylation patterns may differ between biopsies, cfDNA and surgical specimens due to cellular heterogenicity, recent studies using a global approach have demonstrated that the most common cancer-specific DNA methylation markers are consistent between biopsies, cfDNA, and surgical specimens (43–45). We confirmed that bladder cancer cells in cell culture *in vitro* that survive from fresh primary tumors obtained from cystectomy have similar DNA methylation pattern of this primary tumor (46). All of these findings suggest that RS obtained from TURBT, biopsy, or urine sediments may be used as survival predictors for not only preoperative or pretreatment risk stratification, but also to screen in post-cystectomy settings along with clinicopathological features for MIBC patients.

Some DNA methylation biomarkers are also functional markers. The DNA methylation status for two of the probes located in the *OXT1* gene body is positively correlated with OXT1 mRNA expression in MIBC patients. Furthermore, the finding also demonstrates that *OXT1* gene body DNA hypermethylation of the is correlated with its overexpression in MIBC. *OXT1* has been demonstrated as an aggressive oncogene and overexpressed in many types of cancer, including gastric and liver cancers (47, 48), and also been used as a bladder cancer tumor marker of bladder cancer (49, 50). We previously reported that gene body DNA hypermethylation is not only positively correlated with gene expression, specifically for oncogenes, but is also a therapeutic target of DNA methylation inhibitors in cancer (33, 34).

However, there are still some limitations in our study, as the training and test groups both come from the TCGA dataset. An independent patient group is necessary for future validation experiments. Another major limitation is the lack of detailed clinical-pathological variables in the TCGA dataset, such as limited neoadjuvant and adjuvant treatment cases. Using our DNA methylation-based RS and our newly developed nomogram to predict NAC responders *vs.* non-responders should be further validated by a prospective study large cohort.

## Conclusions

In summary, we have constructed a powerful DNA methylation-based classifier that accurately subdivides MIBC patients into long- and short-survival groups. Our study has confirmed that our risk score is independent of most clinical characteristics and has improved prognostic value relative to other clinical features such as T stage and N status. Moreover, we also developed a concise nomogram which only includes age, lymph node status, and RS to predict survival probability of MIBC patients. Taken together, the DNA methylation signature can be used for prediction of MIBC patients independent of clinicopathological features and/or complementary of clinical model, to improve not only preoperative risk classification but also after cystectomy and enhance personalized clinic decision-making.

## Data Availability Statement

The datasets presented in this study can be found in online repositories. The names of the repository/repositories and accession number(s) can be found in the article/**Supplementary Material**.

## Author Contributions

ZX has full access to all data in the study and takes responsibility for the integrity of the data and the accuracy to the data analysis. *Study concept and design:* GL, KS, and ZX. *Analysis and interpretation of data:* ZX, HG, DW, KS, and GL. *Drafting of the manuscript:* ZX, DW, IG, KS, and GL. *Critical revision of the manuscript for clinical implication*: GF, HA, SB, BJ, PG, IG, SD, and GL. *Statistical analysis:* ZX, HG, KS, and GL. All authors contributed to the article and approved the submitted version.

## Funding

This study is supported by Southern California Clinical and Translational Science Institute (SC CTSI, 2019-2020) (GL, SD), the Vicky Joseph Cancer Research Foundation (GL), and (R35 CA209859) (GL) the National Institute of Health, National Cancer Institute (P30 CA014089) (GL and DW).

## Conflict of Interest

DW is a consultant for Zymo Research Corporation (Irvine, CA).

The remaining authors declare that the research was conducted in the absence of any commercial or financial relationships that could be construed as a potential conflict of interest.

## Supplementary Material

Supplementary Figure 1 Construction of the seven-probe classifier. Two unsupervised hierarchical clustering heatmaps showing DNA methylation of the 8,332 and 367 differential DNA methylation probe panels between adjacent-normal and tumor tissues in training set.

Supplementary Figure 2 The correlation between DNA methylation and gene expression of the 19-probe panel based on TCGA bladder tumors and adjacent-normal tissues.

Supplementary Figure 3 Time-dependent ROC curves according to risk score (the prognostic classifier). **(A)** Training set; **(B)** Test set; **(C)** Heatmap of DNA methylation beat values of the seven individual probes in the testing set.

Supplementary Figure 4 3-year and 5-year RS-AUC for each of the 10 training group and testing group ROC curves. **(A)** Training set; **(B)** Testing set.

Supplementary Figure 5 Kaplan-Meier survival analysis for 403 patients (training + testing sets) based on the prognostic classifier stratified by T stage, N stage, and TNM stage. Patients were stratified based on T stage (T1+T2, T3+T4), N stage (N0, N1–3). **(A)** Kaplan-Meier curves for T stage (left panel, N = 369), and further stratified according to the risk score by T1+T2 (middle panel, N = 119), T3+T4 (right panel, N = 250). **(B)** Kaplan-Meier curves for N stage (left panel, N = 360), and further stratified according to the risk score by N0 (middle panel, N = 234), N1–3 (right panel, N = 126). The difference between the two curves were determined using two-sided log-rank test.

Supplementary Figure 6 Association between *DNA* methylation (probes) and gene expression in MIBC. Correlation of *gene* expression with CpG methylation in six probes in pooled MIBC tumors. TCGA RNA-seq data were plotted graphically (x-axis) with DNA methylation beta-values (y-axis) **(A–F)**.

## Abbreviations

MIBC, Muscle-invasive bladder cancer; UBC, urothelial bladder carcinomas; TCGA, The Cancer Genome Atlas; OS, overall survival; LASSO, least absolute shrinkage and selection operator; RS, risk score; ROC, receiver operating characteristic; NMIBC, Non-muscle invasive tumors; NAC, neoadjuvant cisplatin-based chemotherapy; GEO, Gene Expression Omnibus; T, Tumor stage; N, lymph node metastasis; M, distant metastasis; DMP, differentially methylated probe; HRs, Hazard ratios; C-index, the concordance index; TURBT, a transurethral resection of bladder tumor.
