# Supplementary figures and images for: A Novel DNA Methylation Signature as an Independent Prognostic Factor in Muscle-Invasive Bladder Cancer
Source: Front Oncol. 2021 Feb 15;11:614927. doi: 10.3389/fonc.2021.614927 (PMC7917237; doi:10.3389/fonc.2021.614927)

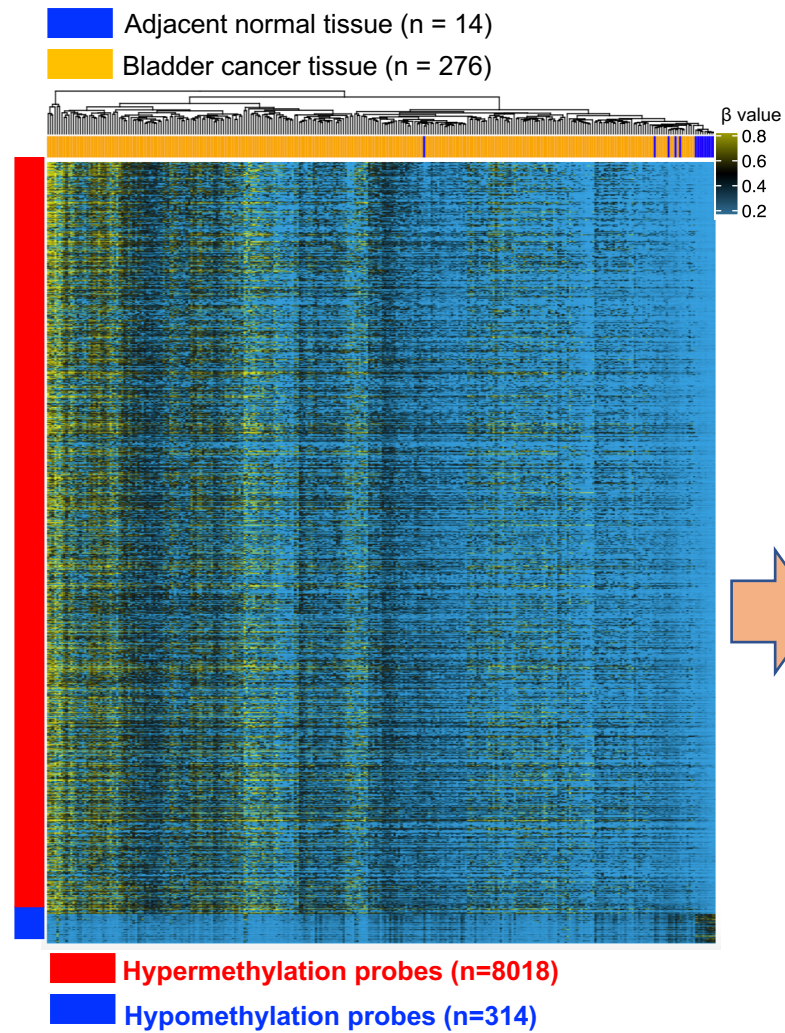

$\log FC > 1.5$  or  $< -1.5$ ,  $FDR < 0.05$

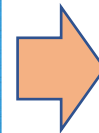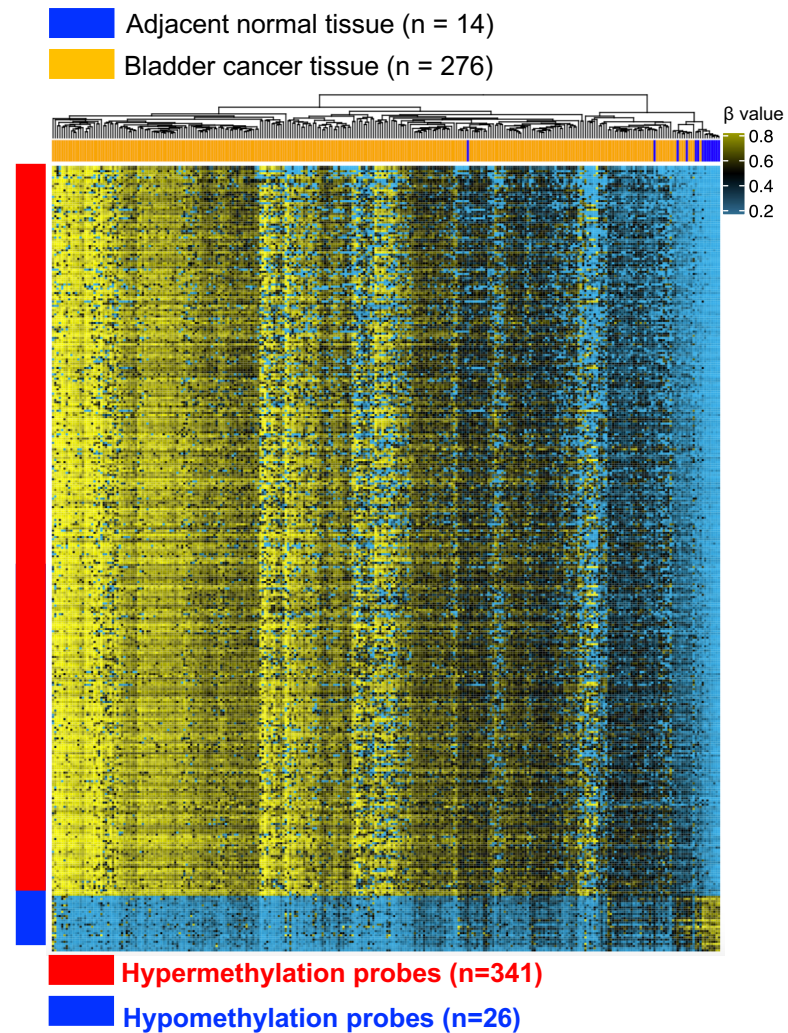

$T-N > 0.4$  or  $< -0.4$

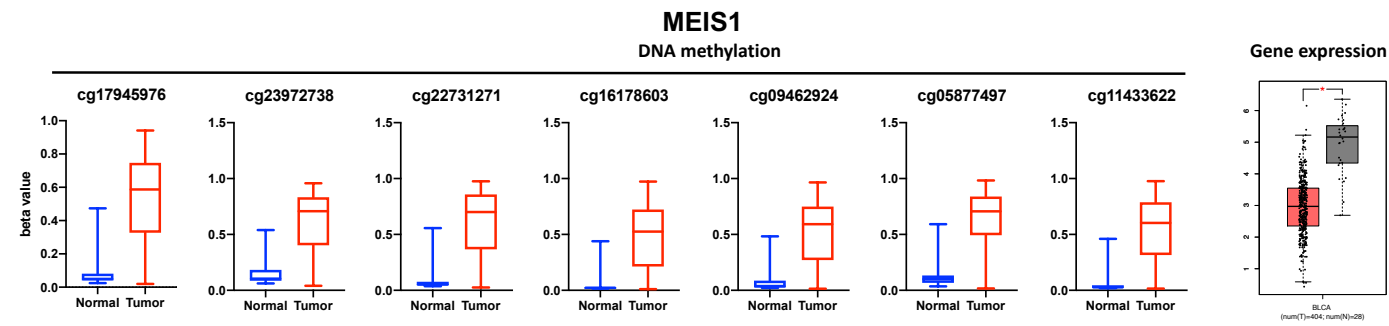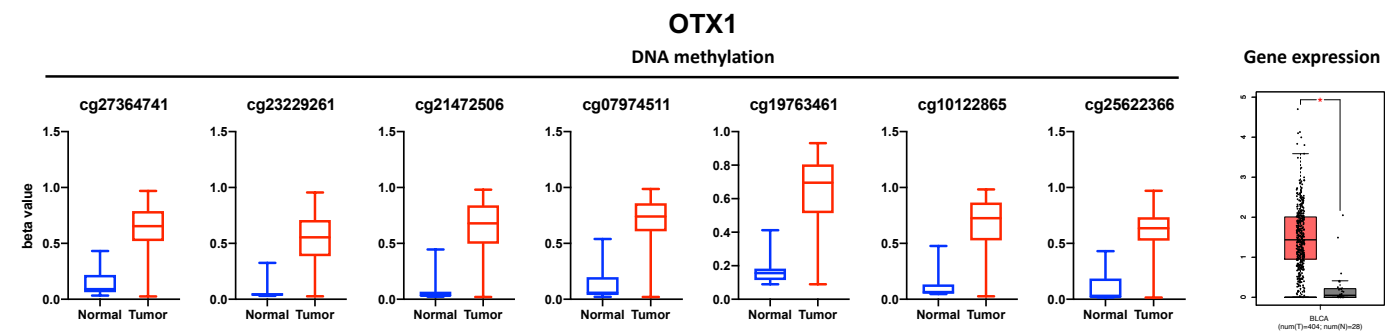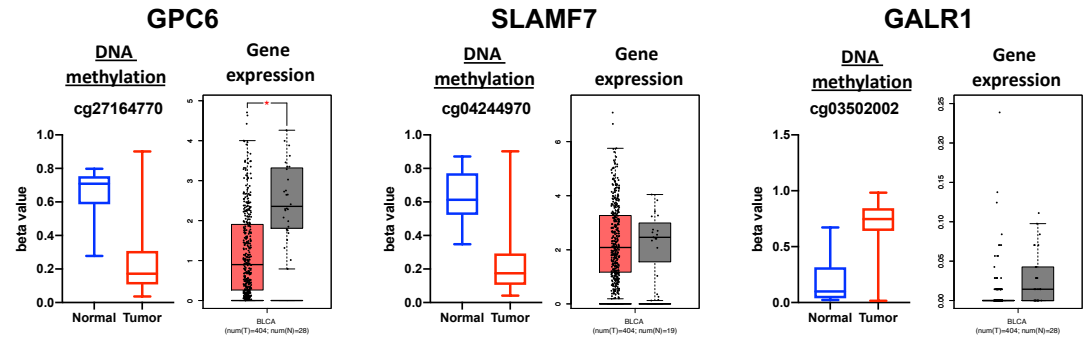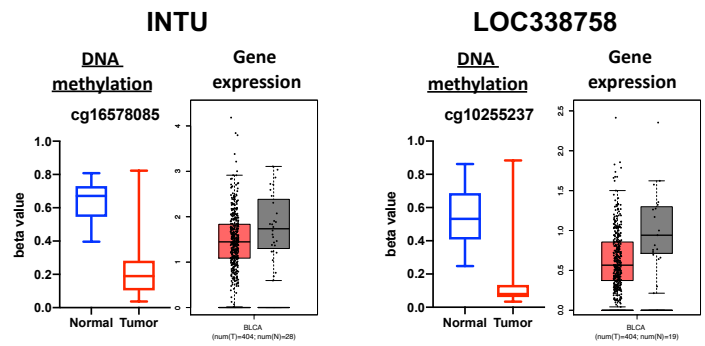

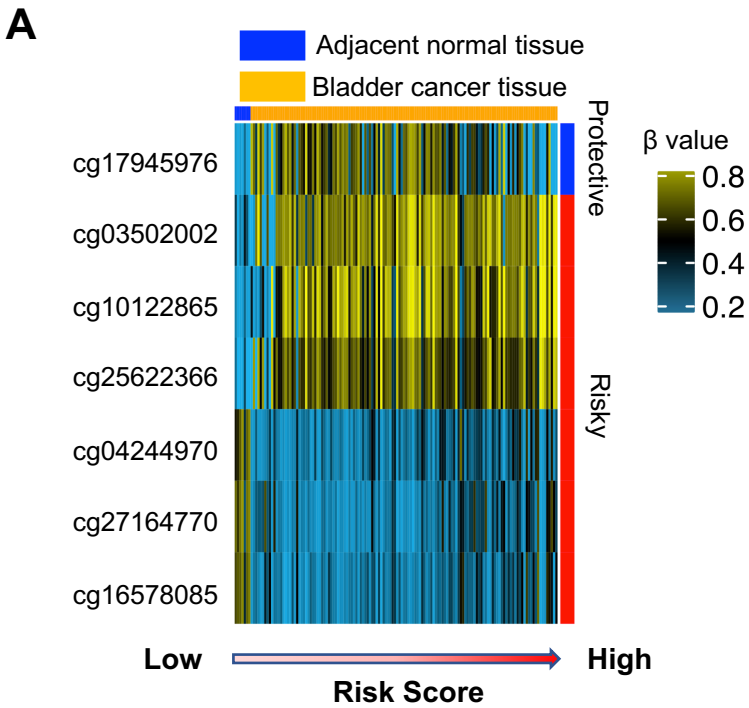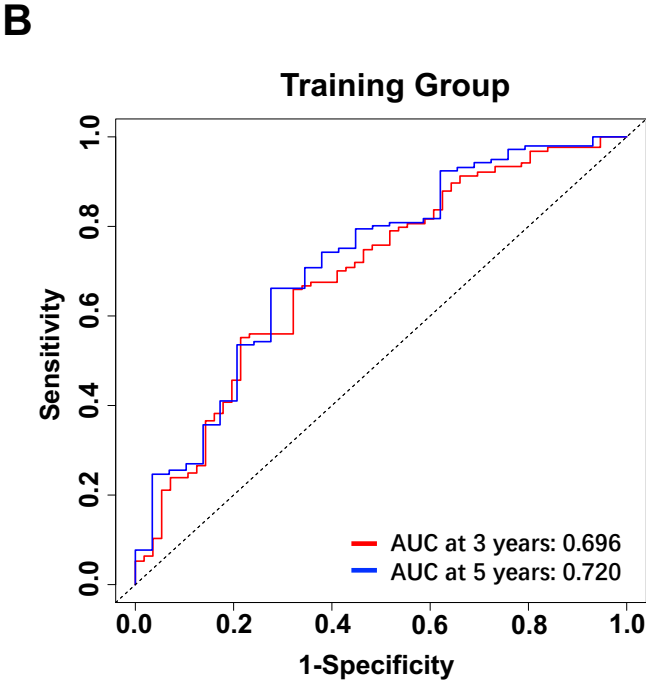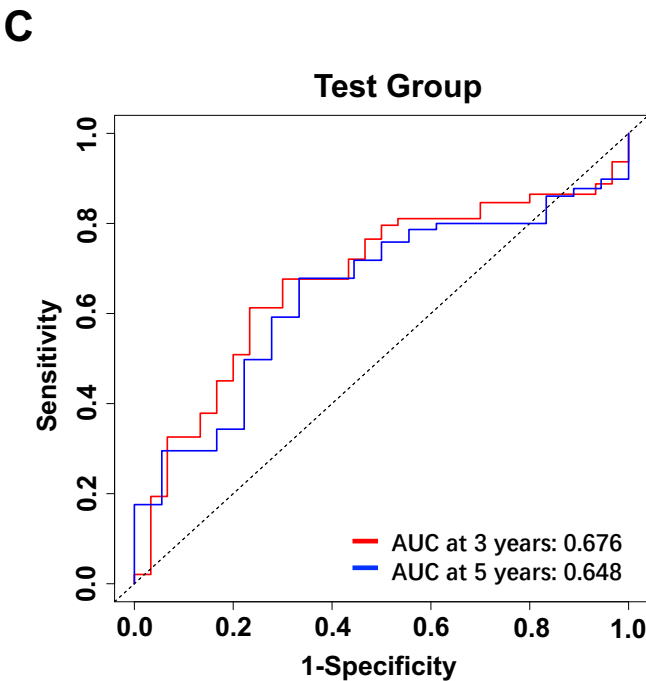

A

3-year AUC

5-year AUC

Training set

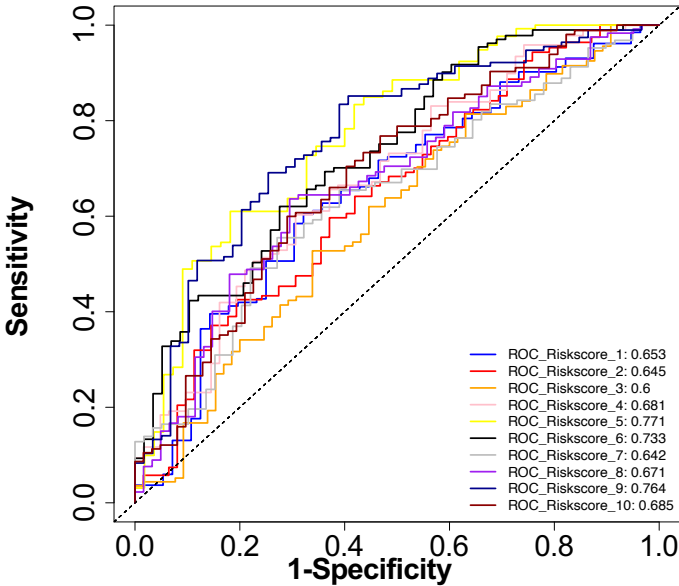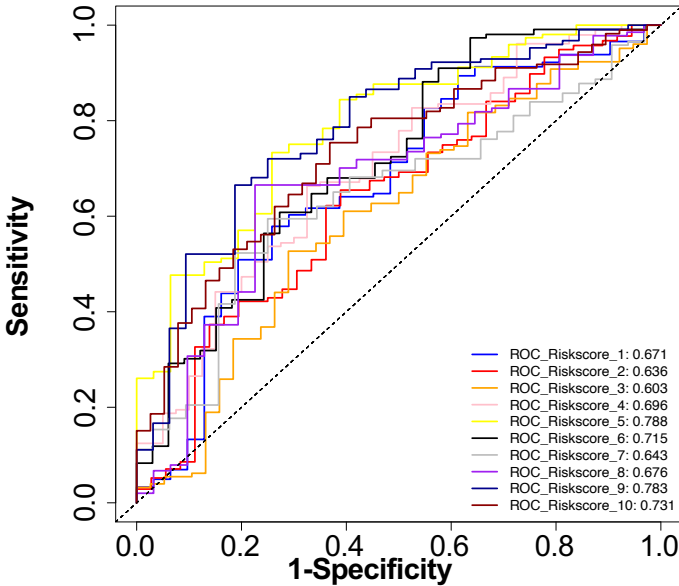

B

Testing set

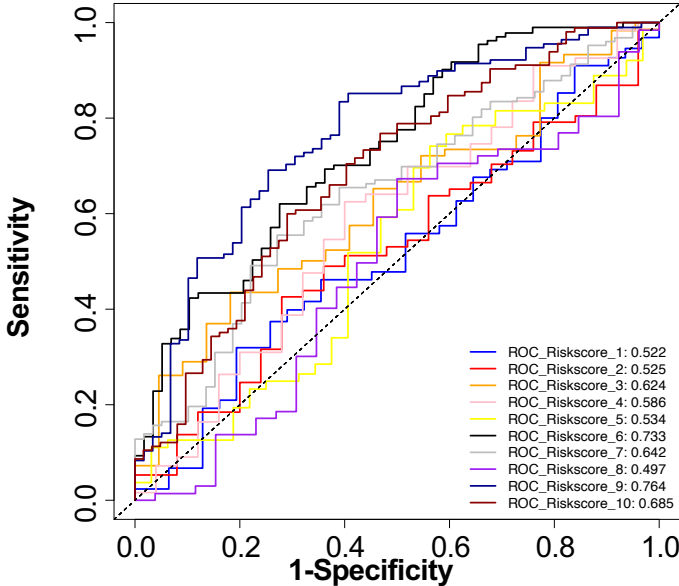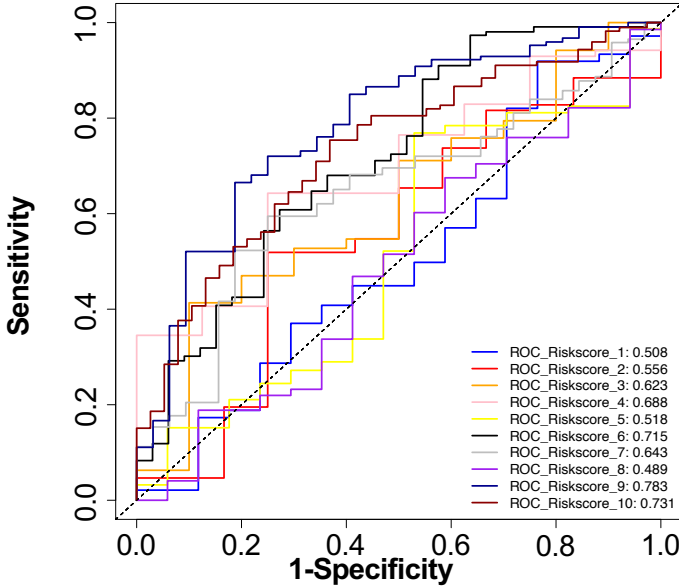

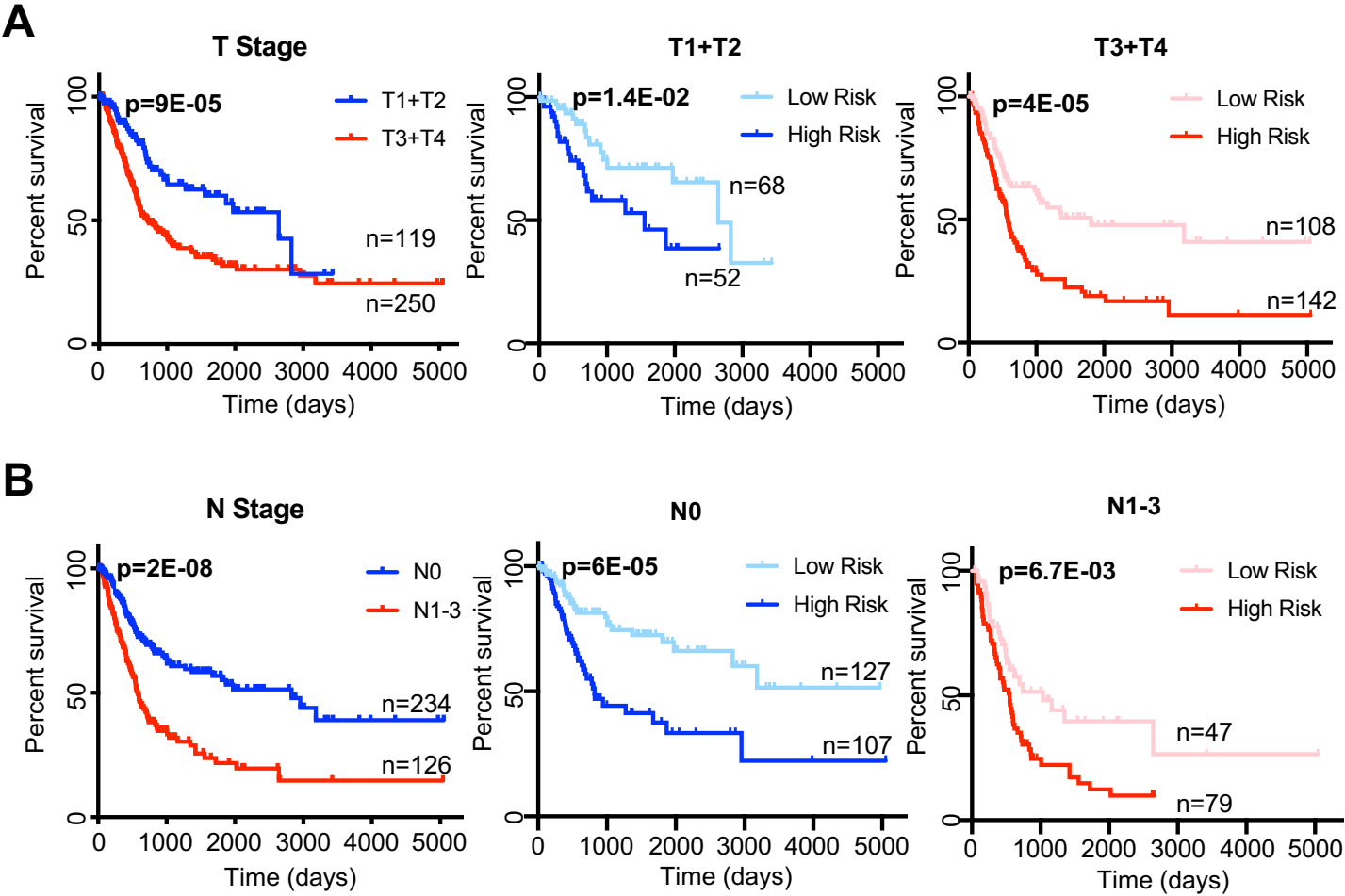

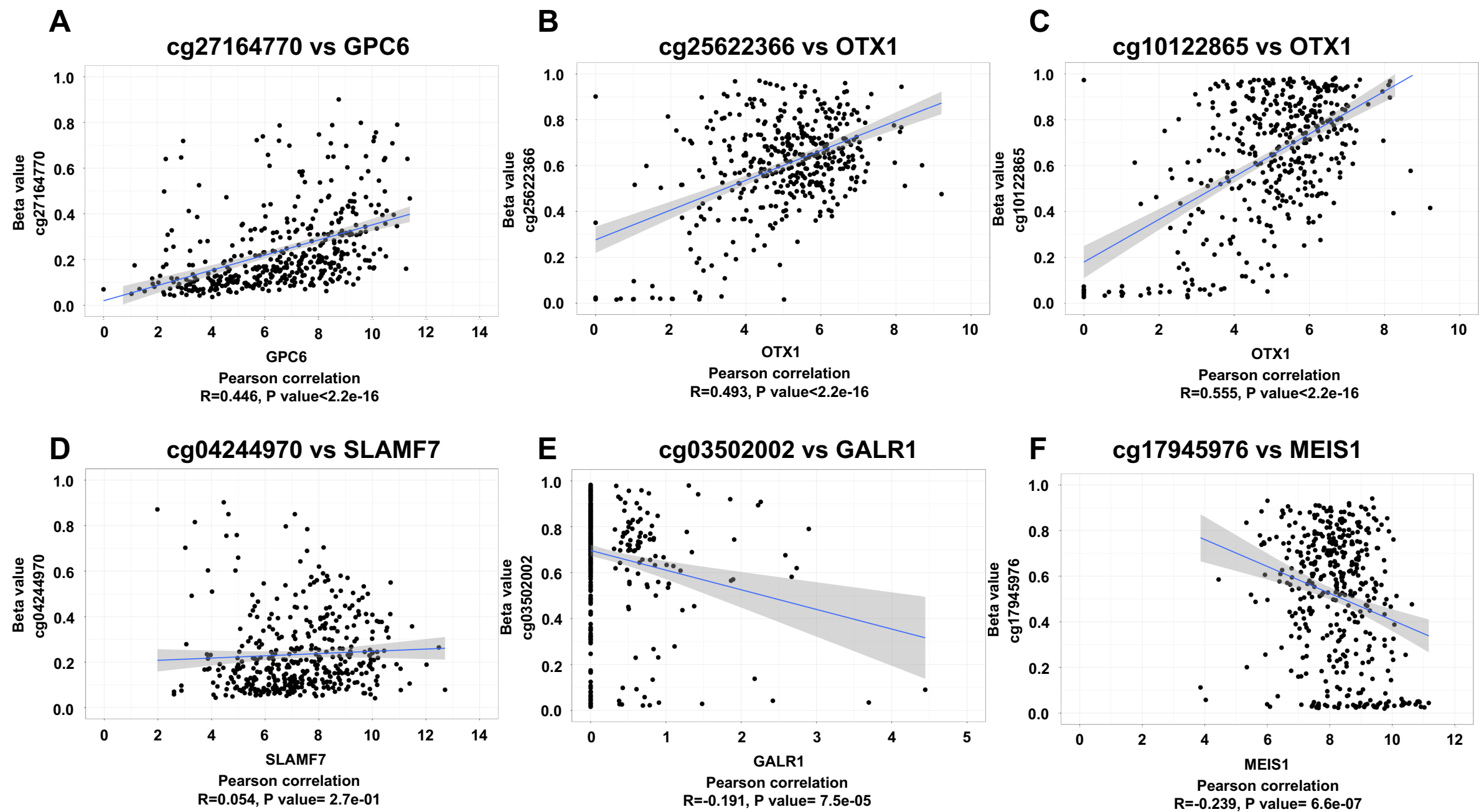

Supplement: Supplementary Figure 1 — Construction of the seven-probe classifier. Two unsupervised hierarchical clustering heatmaps showing DNA methylation of the 8,332 and 367 differential DNA methylation probe panels between adjacent-normal and tumor tissues in training set. [file DataSheet_1.pdf]
