# Supplementary material for: A Novel DNA Methylation Signature as an Independent Prognostic Factor in Muscle-Invasive Bladder Cancer
Source: Front Oncol. 2021 Feb 15;11:614927. doi: 10.3389/fonc.2021.614927 (PMC7917237; doi:10.3389/fonc.2021.614927)
Supplement: Supplementary file 2 [file Table_1.pdf]

**Supplemental Table 1: Repeated sampling for construction of prognostic classifier**

| Replicate | Probe | Gene name | Risk | lasso-coefficient |
|---|---|---|---|---|
| 1 | cg22282405 | TFAP2B | protective | |
| | cg13800209 | MEIS2 | protective | |
| | cg17945976 | MEIS1 | protective | -0.44087198 |
| | cg23972738 | MEIS1 | protective | |
| | cg09462924 | MEIS1 | protective | -0.29896744 |
| | cg16178603 | MEIS1 | protective | |
| | cg17107572 | MEIS1 | protective | |
| | cg22731271 | MEIS1 | protective | |
| | cg09671258 | LHX4 | protective | |
| | cg11433622 | MEIS1 | protective | |
| | cg02970836 | TRNA_Pro | protective | |
| | cg22604316 | DMBX1 | protective | |
| | cg25622366 | OTX1 | risky | 0.59880374 |
| | cg27164770 | GPC6 | risky | 0.30837935 |
| | cg10122865 | OTX1 | risky | 0.9964799 |
| | cg20916523 | VHL | risky | |
| | cg04244970 | SLAMF7 | risky | 0.6591016 |
| | cg19763461 | OTX1 | risky | |
| | cg03502002 | GALR1 | risky | 0.70122593 |
| | cg22691256 | WBSCR17 | risky | |
| | cg16925459 | HDAC9 | risky | |
| | cg16578085 | INTU | risky | 0.45291578 |
| 2 | cg03405909 | PAX6 | protective | |
| | cg22282405 | TFAP2B | protective | |
| | cg09462924 | MEIS1 | protective | |
| | cg23972738 | MEIS1 | protective | -0.00071655 |
| | cg17945976 | MEIS1 | protective | -0.38921262 |
| | cg16178603 | MEIS1 | protective | |
| | cg22731271 | MEIS1 | protective | |
| | cg27164770 | GPC6 | risky | |
| | cg04244970 | SLAMF7 | risky | 0.33858511 |
| | cg03502002 | GALR1 | risky | |
| | cg25622366 | OTX1 | risky | |
| | cg10122865 | OTX1 | risky | |
| | cg07974511 | OTX1 | risky | |
| | cg10255237 | LOC338758 | risky | 0.14903129 |

| | | | | |
|---|---|---|---|---|
| | cg01572460 | FBXL7 | risky | |
| | cg16578085 | INTU | risky | 1.22025144 |
| | cg17467525 | PIK3CD | risky | |
| 3 | cg17945976 | MEIS1 | protective | -0.389212616 |
| | cg09462924 | MEIS1 | protective | |
| | cg23972738 | MEIS1 | protective | |
| | cg22731271 | MEIS1 | protective | |
| | cg10122865 | OTX1 | risky | 0.716554 |
| | cg00582524 | SALL1 | risky | |
| | cg07974511 | OTX1 | risky | |
| | cg21472506 | OTX1 | risky | |
| | cg23229261 | OTX1 | risky | |
| | cg27364741 | OTX1 | risky | |
| | cg03502002 | GALR1 | risky | |
| | cg01532168 | KCNK9 | risky | |
| | cg19763461 | OTX1 | risky | |
| | cg04534765 | GALR1 | risky | |
| | cg05099508 | SPAG6 | risky | |
| | cg27164770 | GPC6 | risky | |
| | cg16578085 | INTU | risky | 1.22025144 |
| | cg01572460 | FBXL7 | risky | |
| | cg10255237 | LOC338758 | risky | |
| | cg22691256 | WBSCR17 | risky | |
| | cg04244970 | SLAMF7 | risky | |
| | cg17467525 | PIK3CD | risky | 0.14903129 |
| | cg13784312 | RAPGEF1 | risky | 0.338585112 |
| 4 | cg09462924 | MEIS1 | protective | -0.4423175 |
| | cg23972738 | MEIS1 | protective | |
| | cg17945976 | MEIS1 | protective | |
| | cg11433622 | MEIS1 | protective | |
| | cg05877497 | MEIS1 | protective | |
| | cg22731271 | MEIS1 | protective | |
| | cg16178603 | MEIS1 | protective | |
| | cg26479667 | LOC285577 | risky | |
| | cg23229261 | OTX1 | risky | 1.4872895 |
| | cg16152136 | FLJ42969 | risky | |
| | cg11329678 | IRX4 | risky | |
| | cg25622366 | OTX1 | risky | |
| | cg10255237 | LOC338758 | risky | 0.2068827 |

| | | | | |
|---|---|---|---|---|
| | cg04244970 | SLAMF7 | risky | 0.3646767 |
| | cg27164770 | GPC6 | risky | 0.2093498 |
| | cg16578085 | INTU | risky | 1.0880277 |
| | cg10122865 | OTX1 | risky | 0.437051 |
| | cg03502002 | GALR1 | risky | 0.2286467 |
| 5 | cg17945976 | MEIS1 | protective | |
| | cg23972738 | MEIS1 | protective | |
| | cg09462924 | MEIS1 | protective | |
| | cg16178603 | MEIS1 | protective | |
| | cg22731271 | MEIS1 | protective | -0.2912206 |
| | cg11433622 | MEIS1 | protective | |
| | cg05877497 | MEIS1 | protective | |
| | cg27164770 | GPC6 | risky | |
| | cg10122865 | OTX1 | risky | 1.7170238 |
| | cg13784312 | RAPGEF1 | risky | |
| | cg03502002 | GALR1 | risky | 1.8290824 |
| | cg04244970 | SLAMF7 | risky | 0.1762511 |
| | cg27364741 | OTX1 | risky | 1.5646846 |
| | cg16578085 | INTU | risky | 0.4746671 |
| | cg05919685 | APCS | risky | 1.6448035 |
| | cg22691256 | WBSCR17 | risky | 1.0274163 |
| 6 | cg23972738 | MEIS1 | protective | |
| | cg22731271 | MEIS1 | protective | -0.3391273 |
| | cg16178603 | MEIS1 | protective | |
| | cg10122865 | OTX1 | risky | 0.02761343 |
| | cg19801921 | LINC00466 | risky | |
| | cg25622366 | OTX1 | risky | |
| | cg19760241 | LHX1 | risky | |
| | cg04244970 | SLAMF7 | risky | |
| | cg23619399 | POU4F1 | risky | 0.10461197 |
| | cg26751356 | SOX1 | risky | 0.25061203 |
| | cg07065111 | SOX11 | risky | 0.35593774 |
| | cg16578085 | INTU | risky | 0.16600471 |
| | cg03502002 | GALR1 | risky | 0.24326837 |
| | cg27164770 | GPC6 | risky | 0.12228677 |
| | cg14754787 | LHX1 | risky | |
| | cg09813525 | PCDH8 | risky | |
| | cg12456714 | BC043001 | risky | |
| | cg10778841 | SORCS3 | risky | |

| | | | | |
|---|---|---|---|---|
| | cg23229261 | OTX1 | risky | 0.71077205 |
| | cg24031355 | SPAG6 | risky | |
| | cg09305680 | UTP23 | risky | 2.10287665 |
| | cg05099508 | SPAG6 | risky | 0.50777308 |
| | cg13784312 | RAPGEF1 | risky | 1.16702333 |
| | cg10255237 | LOC338758 | risky | |
| 7 | cg17945976 | MEIS1 | protective | -0.03875245 |
| | cg09462924 | MEIS1 | protective | |
| | cg05877497 | MEIS1 | protective | |
| | cg22731271 | MEIS1 | protective | |
| | cg27364741 | OTX1 | risky | |
| | cg19763461 | OTX1 | risky | |
| | cg10122865 | OTX1 | risky | |
| | cg23229261 | OTX1 | risky | |
| | cg07974511 | OTX1 | risky | |
| | cg03502002 | GALR1 | risky | |
| | cg25622366 | OTX1 | risky | 0.44835243 |
| | cg09696091 | LOC285577 | risky | |
| | cg21472506 | OTX1 | risky | 0.29204349 |
| | cg04244970 | SLAMF7 | risky | 0.4449185 |
| | cg27164770 | GPC6 | risky | 1.08170948 |
| | cg16578085 | INTU | risky | |
| 8 | cg17945976 | MEIS1 | protective | |
| | cg09462924 | MEIS1 | protective | -0.47527283 |
| | cg11433622 | MEIS1 | protective | |
| | cg16178603 | MEIS1 | protective | |
| | cg23972738 | MEIS1 | protective | |
| | cg22731271 | MEIS1 | protective | |
| | cg05877497 | MEIS1 | protective | |
| | cg14557064 | MIR4277 | risky | |
| | cg07418387 | FMN2 | risky | |
| | cg10216717 | TMEM132C | risky | |
| | cg27164770 | GPC6 | risky | 0.33564875 |
| | cg21472506 | OTX1 | risky | |
| | cg03661929 | LSAMP-AS3 | risky | |
| | cg23229261 | OTX1 | risky | |
| | cg17372657 | AK090593 | risky | |
| | cg01712948 | Ig kappa | risky | |
| | cg16925459 | HDAC9 | risky | |

| | | | | |
|---|---|---|---|---|
| | cg09305680 | UTP23 | risky | |
| | cg09696091 | LOC285577 | risky | |
| | cg04244970 | SLAMF7 | risky | 0.03614674 |
| | cg20168964 | TMEM132D | risky | |
| | cg10255237 | LOC338758 | risky | 0.34414934 |
| | cg22691256 | WBSCR17 | risky | |
| | cg25622366 | OTX1 | risky | 0.39952944 |
| | cg16578085 | INTU | risky | 0.42286426 |
| | cg06035708 | A2BP1 | risky | |
| | cg10122865 | OTX1 | risky | |
| 9 | cg17945976 | MEIS1 | protective | -0.55876773 |
| | cg09462924 | MEIS1 | protective | |
| | cg23972738 | MEIS1 | protective | |
| | cg11433622 | MEIS1 | protective | |
| | cg16178603 | MEIS1 | protective | |
| | cg22731271 | MEIS1 | protective | |
| | cg05877497 | MEIS1 | protective | -0.04098542 |
| | cg10122865 | OTX1 | risky | |
| | cg27164770 | GPC6 | risky | |
| | cg19763461 | OTX1 | risky | 1.19453981 |
| | cg07974511 | OTX1 | risky | |
| | cg03502002 | GALR1 | risky | 1.08471264 |
| | cg10255237 | LOC338758 | risky | |
| | cg16578085 | INTU | risky | 1.2583566 |
| | cg04244970 | SLAMF7 | risky | 0.64382595 |
| | cg25622366 | OTX1 | risky | 1.16212012 |
| 10 | cg09462924 | MEIS1 | protective | -0.2030043 |
| | cg17945976 | MEIS1 | protective | |
| | cg23972738 | MEIS1 | protective | |
| | cg22731271 | MEIS1 | protective | -0.2712798 |
| | cg11433622 | MEIS1 | protective | |
| | cg24031355 | SPAG6 | risky | |
| | cg02671880 | SOX21 | risky | 0.9142339 |
| | cg17911318 | GALR1 | risky | 0.3349362 |
| | cg19763461 | OTX1 | risky | 1.131686 |
| | cg10122865 | OTX1 | risky | 0.6838386 |
| | cg16578085 | INTU | risky | |
| | cg22691256 | WBSCR17 | risky | |
| | cg25622366 | OTX1 | risky | 2.1010153 |

| | | | | |
|---|---|---|---|---|
| | cg10255237 | LOC338758 | risky | |
| | cg27164770 | GPC6 | risky | 0.3815782 |
| | cg04244970 | SLAMF7 | risky | 0.9422421 |

*Probes labeled as red are overlap with identified 19 probes in Table 2
